# Can Serial Dependencies in Choices and Neural Activity Explain Choice Probabilities?

**DOI:** 10.1523/JNEUROSCI.2225-17.2018

**Published:** 2018-04-04

**Authors:** Jan-Matthis Lueckmann, Jakob H. Macke, Hendrikje Nienborg

**Affiliations:** ^1^Research Center Caesar, An associate of the Max-Planck Society, 53175 Bonn, Germany,; ^2^Centre for Cognitive Science, Technische Universität Darmstadt, 64289 Darmstadt, and; ^3^Werner Reichardt Centre for Integrative Neuroscience, 72076 Tübingen, Germany

**Keywords:** choice probability, macaque, perceptual decision-making, sequential dependence, V2

## Abstract

During perceptual decisions the activity of sensory neurons covaries with choice, a covariation often quantified as “choice-probability”. Moreover, choices are influenced by a subject's previous choice (serial dependence) and neuronal activity often shows temporal correlations on long (seconds) timescales. Here, we test whether these findings are linked. Using generalized linear models, we analyze simultaneous measurements of behavior and V2 neural activity in macaques performing a visual discrimination task. Both, decisions and spiking activity show substantial temporal correlations and cross-correlations but seem to reflect two mostly separate processes. Indeed, removing history effects using semipartial correlation analysis leaves choice probabilities largely unchanged. The serial dependencies in choices and neural activity therefore cannot explain the observed choice probability. Rather, serial dependencies in choices and spiking activity reflect two predominantly separate but parallel processes, which are coupled on each trial by covariations between choices and activity. These findings provide important constraints for computational models of perceptual decision-making that include feedback signals.

**SIGNIFICANCE STATEMENT** Correlations, unexplained by the sensory input, between the activity of sensory neurons and an animal's perceptual choice (“choice probabilities”) have received attention from both a systems and computational neuroscience perspective. Conversely, whereas temporal correlations for both spiking activity (“non-stationarities”) and for a subject's choices in perceptual tasks (“serial dependencies”) have long been established, they have typically been ignored when measuring choice probabilities. Some accounts of choice probabilities incorporating feedback predict that these observations are linked. Here, we explore the extent to which this is the case. We find that, contrasting with these predictions, choice probabilities are largely independent of serial dependencies, which adds new constraints to accounts of choice probabilities that include feedback.

## Introduction

During perceptual decisions humans and animals rely on the sensory evidence but also leverage the behavioral context, including their previous decisions (Senders and Sowards, 1952; Verplanck et al., 1952; Seidemann, 1998; Gold et al., 2008; Busse et al., 2011; Akaishi et al., 2014; Fischer and Whitney, 2014; Frund et al., 2014; Abrahamyan et al., 2016; Pape and Siegel, 2016). Such serial dependence of a subject's choices persists with extensive behavioral training (Seidemann, 1998; Gold et al., 2008; Frund et al., 2014), is tuned to the sensory context (Fischer and Whitney, 2014), and is adaptable (Abrahamyan et al., 2016). It may therefore reflect the effect of prior knowledge of statistical regularities in the environment on the perceptual inference process (Helmholtz, 1867; Gregory, 1980; Lee and Mumford, 2003; Yuille and Kersten, 2006; St. John-Saaltink et al., 2016).

Conversely, spiking activity of sensory neurons shows fluctuations on slow timescales up to several seconds. Such slow fluctuations of spiking activity are observed under anesthesia (Tomko and Crapper, 1974; Tolhurst et al., 1983; Ecker et al., 2014; Goris et al., 2014), in awake animals (Ecker et al., 2014), and during task performance (Bair et al., 2001; Rabinowitz et al., 2015; Engel et al., 2016). Rather than noise, these slow fluctuations are increasingly interpreted to also reflect “meaningful intrinsic signals” (Rabinowitz et al., 2015), and can be linked to an animal's cognitive state (Rabinowitz et al., 2015; Engel et al., 2016). It is therefore possible that they, at least partially, represent a signature of the serial dependencies in behavior. Consider, for example, perceptual decision making viewed as probabilistic inference involving task-related feedback to sensory neurons (Haefner et al., 2016). If serial dependencies in behavior reflect prior knowledge in the perceptual inference process (Kok et al., 2014; St. John-Saaltink et al., 2016), such a framework predicts corresponding temporal correlations in the neural activity.

Moreover, this view also has important implications for the observation that the activity of sensory neurons shows correlations with perceptual decisions that are not explained by the sensory stimulus (Logothetis and Schall, 1989; Britten et al., 1996). These trial-by-trial correlations between the activity of sensory neurons and perceptual choices in discrimination tasks are often quantified using “choice probabilities” (CPs; Britten et al., 1996; Dodd et al., 2001; Uka and DeAngelis, 2004; Liu and Newsome, 2005; Shiozaki et al., 2012; Nienborg and Cumming, 2006, 2014). CPs have been used to gain insights into decoding strategies (Haefner et al., 2013; Pitkow et al., 2015; Clery et al., 2017), and can result from both feedforward and feedback mechanisms (Nienborg et al., 2012; Cumming and Nienborg, 2016). Importantly, the decision-related feedback mechanisms that have been invoked to contribute to CPs (Engel et al., 2015; Nienborg and Roelfsema, 2015; Wimmer et al., 2015; Haefner et al., 2016) originate directly from the decision variable that determines the decision. Because the decision variable, which might be implemented in a higher-order decision circuit (Wimmer et al., 2015), is the only determinant of the decision, the influence of past decisions needs to be reflected at this level. Similarly, if the sensory neurons receive feedback from this decision variable, the influence of past decisions that affects the current decision will be reflected at the level of these sensory neurons. In these models this therefore predicts that in the presence of serial dependencies of behavior, a component of CPs is explained by past decisions.

**Figure 1.**
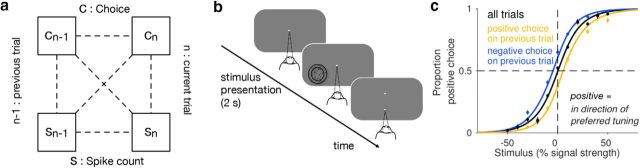
Conceptual framework, task and psychophysical performance. ***a***, Schematic showing all possible statistical dependencies between choices and spike counts on the preceding and current trial. ***b***, After the monkey acquired fixation on a central fixation point the stimulus was presented for a fixed duration of 2 s, followed by two choice targets above and below the fixation point. After a saccade to the correct choice target, the monkey was rewarded with a liquid reward. ***c***, The average psychophysical performance of both monkeys across all sessions (*n* = 75) is shown (black). For each session, a positive and negative choice was defined as a choice toward the neuron's preferred or null disparity (near or far), respectively. Note the horizontal shift of the psychophysical curves with respect to each other when they were computed separately for trials preceded by a positive choice (yellow) or negative choice (blue), indicating a systematic bias introduced by the choice on the preceding trial. Data points are binned averages across sessions. Error bars are SEs. Solid lines represent the mean fits (cumulative Gaussians) across sessions.

Here, we test this prediction in single-unit recordings from macaque visual area V2 while two animals performed a disparity discrimination task. First, we explore the effect of choice history on behavior and on neural activity using generalized linear models (GLMs). Consistent with previous studies (Senders and Sowards, 1952; Verplanck et al., 1952; Seidemann, 1998; Gold et al., 2008; Busse et al., 2011; Akaishi et al., 2014; Fischer and Whitney, 2014; Frund et al., 2014; Abrahamyan et al., 2016; Pape and Siegel, 2016) we find substantial predictive effects of choice history on the animals' choices, with choice-history having a higher CP than single neurons in V2. We also identify strong temporal correlations in spiking activity, as well as a modest predictive effect of choice history on neural spiking activity. We then investigate which covariates of the previous trial are statistically significant predictors of choices and spiking in the next trial (Fig. 1*a*). Finally, we use semipartial correlation analysis to examine the role of choice history on CPs. In contrast with the above prediction, we find that the serial dependencies of choices and spiking activity cannot explain CPs. Rather, they reflect two largely independent parallel temporal processes. This suggests that the feedback contribution to CPs is less pronounced or reflects a more complex process than previously thought.

## Materials and Methods

We performed novel analyses of previously published data (Nienborg and Cumming, 2009). The details of the stimulus, and the behavioral and neurophysiological procedures have been described in detail previously (Nienborg and Cumming, 2009). Here, we briefly summarize the experimental methods.

### 

#### 

##### Electrophysiology.

All procedures were performed in accordance with the U.S. Public Health Service policy on the humane care and use of laboratory animals, and all protocols were approved by the National Eye Institute Animal Care and Use Committee. Extracellular activity from disparity-selective V2 single units was recorded while two male monkeys (*Macaca mulatta*) performed a coarse disparity discrimination task. For each session the signal disparities (one “near”, one “far”) were tailored to the tuning preference of the simultaneously recorded neuron such that one disparity was close to the neuron's preferred disparity and the other at a trough of the neuron's tuning curve.

##### Behavioral task.

Once the animals acquired fixation, the stimulus was presented for a fixed duration of 2 s, followed by the presentation of two choice targets 3° above or below the fixation marker. If the monkeys made a saccade to the correct choice target, they received a liquid reward.

##### Visual stimuli.

The stimuli were circular dynamic random dot patterns consisting of a disparity-varying center (typically 2–4° in diameter) surrounded by an annulus at zero disparity (1–2° wide). The center disparity changed randomly on each video frame (96 Hz frame rate) chosen from an evenly spaced set of disparity centered around 0° disparity, encompassing the tuning preferences of the recorded neuron. For “no-signal trials” (randomly interleaved) all disparities were drawn from a uniform distribution of probabilities. On other trials (“signal” trials) we increased the probability of occurrence (typically by 25, 12.5, and 6%) of one disparity (the “signal disparity”), whereas for the remaining video frames the disparities were drawn from the same uniform distribution as used for the no-signal trials. For each recording session we used two signal disparities, one near disparity and one far disparity, which approximated the neuron's preferred and null disparities.

##### Analysis.

To avoid that neural variability on no-signal trials (i.e., for which disparities occurred with equal probability) reflected systematic choice-dependent stimulus differences, we corrected spike counts on no-signal trials for stimulus-induced variability as described previously (Nienborg and Cumming, 2009). The analyses here were performed on the same core dataset of *n* = 76 neurons as used by Nienborg and Cumming (2009), but restricted to trials which were immediately preceded by at least one complete trial. This reduced the number of included trials per neuron and we required an additional inclusion criterion of at least four near and far choices each for the no-signal trials, which 75/76 neurons passed. These represent the main dataset analyzed here. In all but four sessions, only one unit was recorded per session.

##### Choice correlations.

We converted choice prediction performance (analogous to CP computed as the area under a receiver-operating curve (ROC); cf. Britten et al., 1996) into choice correlations (Haefner et al., 2013; Pitkow et al., 2015).

Based on the results from Haefner et al. (2013), we calculated choice correlations as *corr_xy_* = $\frac{\alpha }{\sqrt{1+\alpha^{2}}}$ with α = $\frac{\alpha }{\sqrt{1+\alpha^{2}}}$. Pitkow et al. (2015) used a linear approximation to this quantity.

##### Statistical modeling.

We fit GLMs (Park et al., 2014) to predict neural spiking and behavioral decisions from experimental covariates via maximum likelihood estimation using MATLAB (MathWorks) to determine the weights (β) for each of the covariates. Separate fits were performed for each neuron. For predicting choices, we used a probit-GLM with lasso regularization (biasing weights toward 0). The value for the regularization hyper-parameter λ was chosen such that the cross-validated prediction performance across the population was maximized (λ = 0.025). To fit single-trial spike counts (i.e., total number of spikes in a 2 s trial) from experimental covariates, a Poisson GLM with exponential nonlinearity was used. For some of the models predicting spike counts, we included a Gaussian process (Rabinowitz et al., 2015) to model slow fluctuations in spike counts (Rasmussen and Williams, 2006; Park et al., 2015). We used a squared exponential kernel for the Gaussian Process prior, exp( − (*r*/τ)^2^). The value for its hyper-parameter τ was chosen such that the cross-validated prediction performance across the population was maximized (τ = 35 trials). We did not use a sparsity penalty when predicting spike counts, because including it did not lead to an improvement in prediction performance. To ensure that estimates of choice- and spike-prediction performance were free of bias from overfitting, we cross-validated the model fits (Hastie et al., 2009). The models were fit to all signal trials of a session and tested only on no-signal trials. For each session, the sign of the choices was defined by the preferred disparity of the simultaneously recorded neuron. Choices of the preferred and null disparity of a neuron were defined as positive and zero, respectively. We typically evaluated model performance by including parameters cumulatively (see Results) to test different hypotheses. Because we cross-validated the models, including more parameters does not necessarily improve performance. In addition, lasso-regularization biases weights on nonpredictive covariates to 0, which means that not all covariates contribute to the prediction. However, we explored all permutations of covariates to verify that there was no combination of covariates that substantially exceeded the prediction performance of the model that was fit to all covariates.

To predict behavioral choices, we used a generalized linear classification model with a probit function as link function (Hastie et al., 2009). This means that a weighted sum of covariates was computed and then passed through the probit function (i.e., the cumulative distribution function of a Gaussian) to predict the probability of a positive choice (*C_n_* = 1): for example, when predicting choice from the current stimulus *S_n_* and the current choice spike count, *m_n_* = β*_o_* + β*_C_n__C_n_* + β*_S_n__ S_n_* and Prob(*C_n_* = 1) = normcdf(*m_n_*). We quantified GLM performance on cross-validated data by fitting the model on signal trials and computing the area under the ROC of the output of the GLM on no-signal trials. The experimental covariates (*z*-scored before fitting) for GLM fits predicting behavior were as follows:

*C_n_*_-1_, choice on the preceding trial
*T_n_*_-1_, target on the preceding trial
*W_n_*_-1_, whether the preceding trial was rewarded (win)
*I_n_*, stimulus (image) on the current trial
*S_n_*, spike count on the current trial
*S_n_*_-1_, spike count on the preceding trial (the stimulus-induced effect is regressed out).

We refer to the covariates, *C_n_*_-1,_ *T_n_*_-1,_ *W_n_*_-1_ together as choice history, abbreviated as *H_n_*_-1_. We quantified the decrease in psychophysical performance attributable to choice history using the approximation derived by Frund et al. (2014) as $\frac{\pi }{16}\sigma^{2}$ where σ^2^ is the choice history-induced variance of the behavior. To control for the effect of the stimulus on the previous trial, *I_n_*_-1_, on the previous spike count, S_n-1_, we regressed out the stimulus-induced effect. To this end we used the residual spike count obtained after linearly regressing *S_n_*_-1_ on *I_n_*_-1_ (without a constant intercept term; including one does not affect our results significantly).

The performance of the GLM-fits predicting spike counts was evaluated by computing the Pearson correlation coefficient between the predicted and measured spike counts on no-signal trials. The covariates for GLM fits predicting spike counts were as follows:

*C_n_*, choice on the current trial
*C_n_*_-1_, choice on the preceding trial
*T_n_*_-1_, target on the preceding trial
*W_n_*_-1_, whether the preceding trial was rewarded (win)
*I_n_*, stimulus (image) on the current trial
*S_n_*_-1_, spike count on the preceding trial (the stimulus-induced effect is regressed out)
SF, slow fluctuation across multiple trials.

In addition, for each unit, we fit a constant intercept term which controls the firing rate of the unit.

For the Poisson GLMs predicting time-resolved neural activity additional covariates were used. To account for the temporal structure of firing within trials, we introduced peristimulus time histogram (PSTH) basis functions, as well as time-varying features for *C_n_*, *C_n_*_-1_, *T_n_*_-1_, and *W_n_*_-1_. To find PSTH basis functions we performed PCA on the PSTHs after correcting for response latency, across all units. Two basis functions were sufficient to describe >99% of the variance of the PSTHs of all units across all stimuli. These two basis functions (minus the mean PSTH of the respective unit) were included in the GLM fit for each unit. The covariates for GLM fits predicting time-resolved spike counts were as follows:

*C_n_*, choice on the current trial (4 predictors)
*C_n_*_-1_, choice on the preceding trial (4 predictors)
*T_n_*_-1_, target on the preceding trial (4 predictors)
*W_n_*_-1_, whether the preceding trial was rewarded (4 predictors)
*S_n_*_-1_, spike count on the preceding trial (the stimulus-induced effect is regressed out)
*I_n_*, stimulus (image) on the current trial
PSTH, PSTH basis functions (2 predictors)
Intercept term (per unit).

Each trial was split into 20 bins of 100 ms each (which corresponds to the resolution of the PSTH basis functions included). For choice and choice history covariates, we included four predictors each into the design matrix of the full model: those four predictors were chosen to distinctly account for effects between 0 and 500, 500 and 1000, 1000 and 1500, and 1500 and 2000 ms of trials. The GLMs were fit on signal trials and the resulting weights used to predict the time-varying spike counts (100 ms resolution) for each no-signal trial.

##### Independent observer model.

To verify that the measured behavioral strategies by the animals were not confounded by the weak intertrial dependence in stimulus induced by pseudorandomization, we also analyzed the responses of a model without choice history (independent observer model). We first fitted psychometric functions with a cumulative Gaussian to match the model's performance for the stimulus on the current trial to the psychophysical performance of the animals. Next, we generated model responses based on the stimulus on the current trial assuming a binomial distribution defined by the model parameters of the psychometric functions. Therefore, in this simulation, the stimulus sequence and the association between current stimulus and response were exactly as in the actual experiment, but choices were not influenced by experimental history. When we performed our analysis on these data, it did not identify any significant or systematic components in the behavioral strategies (see Fig. 3*b–c*). For this analysis, one session was excluded for having less than four near and four far choices on no-signal trials due to random sampling.

##### Semipartial correlations.

We computed correlations between the residuals of the spike counts obtained after linearly regressing *S_n_* on *H_n_*_-1_, and the animals' choices (for no-signal trials). In contrast to the above GLM-analysis, no cross-validation was used when calculating residuals for the correlation analysis. The semipartial correlation is then defined to be the correlation between the choices and the spike-count residuals.

The absolute value of a semipartial correlation constitutes a lower bound (Cohen et al., 2003) on the absolute values of partial correlations between spike counts and choices, i.e., the correlation one would obtain after removing the effects of choice history from both. In the case of partial correlations, we decompose choices *c* into a predicted part *ĉ* and a choice residual *c̃*, such that *c* = *ĉ* + *c̃*. Similarly, for spike counts, *s* = *ŝ* + *s̃*. The absolute value of the semipartial correlation corr(*c*,*s̃*) is a lower bound to the partial correlation corr(*c̃*,*s̃*), i.e., |corr(*c̃*,*s̃*)|≤|corr(*c̃*,*s̃*)|: this can be seen to hold true writing the inequality as follows:

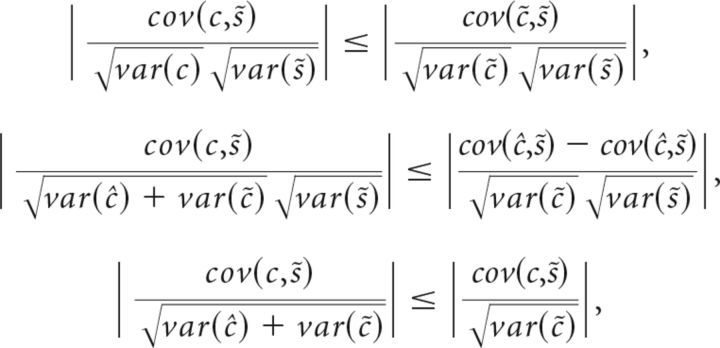
 because cov(*ĉ*,*s̃*) = 0.

##### Autoregressive models.

We performed simulations to test the statistical power of our analyses. Specifically, we defined two autoregressive models that are compatible and two models that are incompatible with our conclusions. In the compatible models (CMs) CPs arise from instantaneous feedforward (CM 1) or postdecision feedback (CM 2) correlations, whereas in the incompatible models (ICMs) they result from the effect of preceding choices on current neural activity (ICM 1) or of preceding neural activity on current choices (ICM 2), respectively.

CM 1:

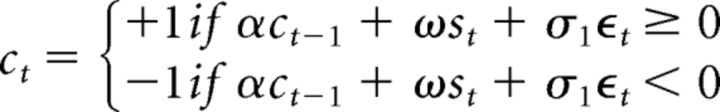


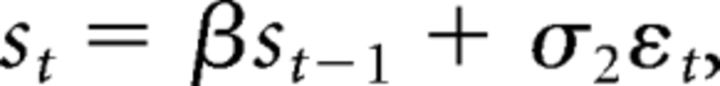
 where ϵ and ε are Gaussian noise, *c_t_* and *s_t_* the choice and spike count on trial *t*, respectively. We note that CM 1 can be easily extended to populations of (potentially) correlated neurons: in that case, the term ω*s_t_* instead consists of the average activity of a population of neurons, and the weights and the strength of the “decision noise” σ_1_ would have to be adjusted such that the model remains consistent with the observable single-neuron statistics. However, as our analyses are based on (temporal) correlations between single-neuron spiking and choices, our results hold regardless of the population size and the size of these noise correlations.

CM 2

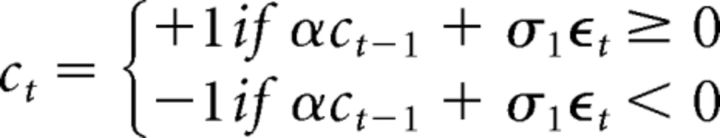


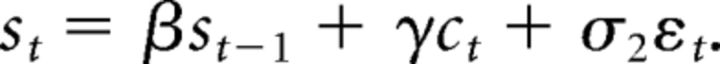
 ICM 1

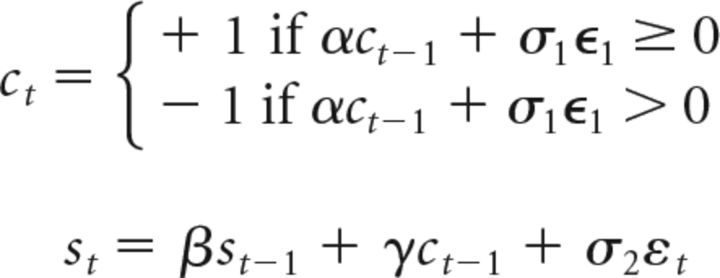
 ICM 2

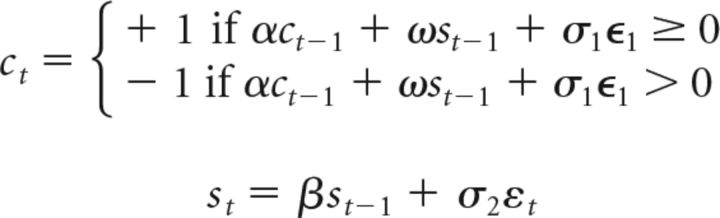
 In analogy to CM 1, ICM 2 can be easily extended such that ω*s_t_*_−1_ is replaced by a population of weakly correlated neurons, and our results are robust to population size and the size of these noise correlations within empirical ranges.

For each of the 75 units from our dataset, we fit models (i.e., α, β, γ, σ_1_, σ_2_, or α, β, ω, σ_1_, σ_2_, respectively) using empirically observed statistics: CPs, auto-covariance between choices on subsequent trials, and auto-covariance between spike counts on subsequent trials. Models were fit using the CMA-ES algorithm (evolution strategy using covariance matrix adaptation; Hansen and Ostermeier, 2001).

After finding model parameters of best fit for each unit, we simulated the same number of trials as there were no-signal trials for this in our dataset. This was done repeatedly (500 repetitions). For each repetition we then computed the semipartial CP (regressing out spike history and choice history) and performed a one-sample *t* test with the null hypothesis that the mean semipartial CP across the population of units is equal 0.5. This recapitulates our semipartial correlation analysis in simulated data.

## Results

**Figure 2.**
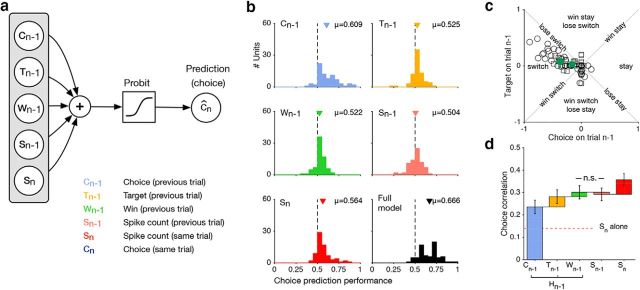
Choice history is systematically associated with the monkeys' current behavior. ***a***, We use a GLM to analyze the statistical effect of different covariates on the monkeys' choices for *n* = 75 sessions (*n* = 41 for Monkey 1; *n* = 34 for Monkey 2). ***b***, The choice prediction performance (aROC; see Materials and Methods) evaluated only for no-signal trials for different covariates: the choice on the previous trial (*C_n_*_-1_, mean CPP 0.609, *p* < 10^−8^); target on the previous trial (*T_n_*_-1_, mean CPP 0.525, *p* < 0.001); whether the preceding trial was rewarded (win, *W_n_*_-1_, mean CPP 0.522, *p* < 0.01), the spike count on the current trial (*S_n_*, mean CPP 0.564, *p* < 10^−7^); the spike count on the previous trial (*S_n_*_-1_, mean CPP 0.504, *p* = 0.378). For the full model, CPP is 0.666, *p* < 10^−10^. The *p* values indicate whether CPPs were significantly different from chance performance (0.5) using Wilcoxon signed rank tests. ***c***, The weights for the target on the previous trial (*T_n_*_-1_) are plotted against those of the previous choice (C_n-1_) for all *n* = 75 sessions (circles: Monkey 1, *n* = 41; squares: Monkey 2, *n* = 34). Green symbols represent means across all sessions for each monkey. ***d***, The mean choice prediction performance converted to correlation coefficients (choice correlation; see Materials and Methods) are plotted for models incorporating the variables on the *x*-axis cumulatively. From left to right the variables used are as follows: (*C_n_*_-1_); (*C_n_*_-1_, *T_n_*_-1_); (*C_n_*_-1_, *T_n_*_-1_, *W_n_*_-1_); (*C_n_*_-1_, *T_n_*_-1_, *W_n_*_-1_, *S_n_*_-1_); (*C_n_*_-1_, *T_n_*_-1_, *W_n_*_-1_, *S_n_*_-1_, *S_n_*). The height of each bar reflects the incremental improvement of the model prediction caused by the variable plotted on the *x*-axis. Significant increments are caused by *C_n_*_-1_ (*p* < 10^−8^), *T_n_*_-1_ (*p* < 10^−5^), *W_n_*_-1_ (*p* = 0.046), *S_n_* (*p* < 10^−4^), but not additionally by *S_n_*_-1_ (*p* = 0.954), Wilcoxon signed rank tests for all. The horizontal dashed line marks the value for a model incorporating only *S_n_*. Error bars are ±1 SE; Colors as in ***b***.

We analyzed behavioral and neuronal data from 75 disparity selective single units in V2 recorded from two monkeys performing a coarse disparity discrimination task as described previously (Nienborg and Cumming, 2009). In this task, schematically summarized in Figure 1*b*, the fixating monkeys were presented with a dynamic random dot pattern positioned inside the receptive field of the simultaneously recorded neurons for a fixed 2 s duration. The stimulus was a circular random dot stereogram defining a central circular disk surrounded by an annulus. The monkeys' task was to judge whether they perceived the central disk as protruding (near) or receding (far) relative to the surround by making a saccade to one of two choice targets. Correct choices were rewarded. The disparity of the stimulus center was spatially uniform on each video frame but changed probabilistically between frames to control the difficulty of each trial (see Materials and Methods). On a subset of trials, defined as no-signal trials, disparities were drawn from a uniform distribution centered on 0 degree disparity. On these trials there was no correct answer and the monkeys were rewarded randomly on 50% of the trials. For discrimination tasks, the dependence of an observer's choices on the stimulus can be captured by a sigmoidal function (psychometric function). For example, for the disparity discrimination task used here, the psychometric function maps the probability of a far choice as a function of the far signal in the stimulus (Fig. 1*c*). Note that for each session we define the sign of the stimulus based on the tuning preference of the simultaneously recorded neuron: positive and negative values correspond to signal strength at the neuron's preferred and nonpreferred disparity, respectively. Typically, the psychometric function includes a stimulus independent term to account for an observer's bias. As shown in Figure 1*c*, this psychophysical bias differed between trials following a choice to the preferred (positive) and nonpreferred (negative) disparity target. To examine any systematic effect of previous choices on the decision in the current trial in more detail, we used a statistical model that, in addition to the stimulus, took into account the influence of previous choices on the animal's present choice (Fig. 2*a*). In this model, the psychophysical bias-term was different for each trial, and depended linearly on the recent experimental history (Seidemann, 1998; Gold et al., 2008; Frund et al., 2014).

### Predictive effect of choice history on choice

**Figure 3.**
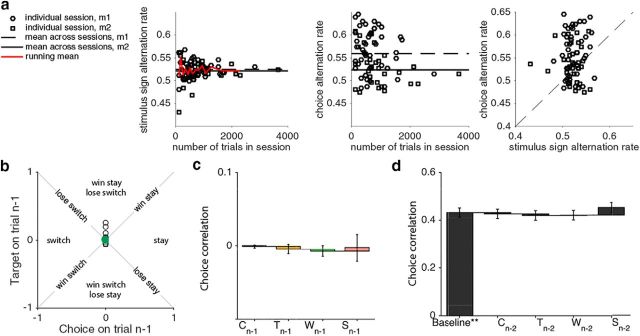
Controls. ***a***, Stimulus statistics and choice alternation rate. Left, We plot the probabilities of alternation of the stimulus sign for each session as a function of session duration (in number of trials) across all sessions (red line: running mean alternation rate across all sessions). To explore whether the animals' strategy depended on the duration of the session we also plot the animal's choice-alternation rate as a function of session duration (middle), and stimulus sign alternation rate (right) and do not observe a systematic relationship. ***b***, The weights of the analysis of the independent observer model for the preceding choice (*C_n_*_-1_) are plotted against those for the preceding target (*T_n_*_-1_) for *n* = 74 sessions. For both covariates the weights do not significantly differ from 0 (*C_n_*_-1_: mean = 0.000, *p* = 1.0; *T_n_*_-1_: 0.006, *p* = 0.297; Wilcoxon signed rank tests). ***c***, ***d***, We compared the choice prediction performance (in choice correlation) as in Figure 2*d*: the height of the bars corresponds to the incremental improvement by the corresponding covariates on the *x*-axis. ***c***, The ability to predict choices based on *C_n_*_-1_, *T_n_*_-1_, *W_n_*_-1_, or *S_n_*_-1_ is at 50% chance level, corresponding to 0 choice correlation (covariates vs 0: all *p* > 0.05; Wilcoxon signed rank tests). Format and colors in ***b*** as in Figure 2*c*, in ***c*** as in Figure 2*d*. ***d***, The effect of choice history beyond the preceding trial is small. For this analysis, we included only 50 sessions (30 for Monkey 1; 20 for Monkey 2), which met our inclusion criteria: at least four near and four far choices on no-signal trials, and selecting only those segments with at least three consecutive trials completed. The baseline model includes the covariates pertaining to the preceding trial (*C_n_*_-1_, *T_n_*_-1_, *W_n_*_-1_, *S_n_*_-1_). Adding the choice on trial *n*-2 (*C_n_*_-2_), yields a nonsignificant increase (*p* = 0.405; Monkey 1: *p* = 0.501; Monkey 2: *p* = 0.583; Wilcoxon signed rank tests). Similarly, the covariates *T_n_*_-2_, *W_n_*_-2_, and *S_n_*_-2_ do not increase the choice correlation. Error bars are ± 1SE.

Conceptually, the contribution of the previous choices can be viewed as the subject's bias that changes from trial to trial depending on the choice history. We used penalized maximum likelihood estimation (Hastie et al., 2009) to fit the weights for both the stimulus and the effect of previous choices. We characterized the monkeys' strategy, i.e., how they were influenced by the previous choice and how this influence depended on whether the previous choice was rewarded, with two covariates, the previous target (*T_n_*_-1_) and the previous choice (*C_n_*_-1_). Different strategies can be identified by plotting the weights of the model for *C_n_*_-1_ against those for *T_n_*_-1_ (Fig. 2*c*; Frund et al., 2014; Abrahamyan et al., 2016). The weight for the previous choice (*C_n_*_-1_, abscissa) quantifies in which direction, on average, the monkeys are influenced by the previous choice: positive values indicate that they tend to repeat their previous choice (a near choice following a near choice, or far following far), whereas negative values indicate that they tend switch their answer (a near choice following a far choice or vice versa). In contrast, the weight for the previous target (*T_n_*_-1_, ordinate, i.e., the choice that would have been rewarded; note that if the preceding trial had no signal *T_n_*_-1_ is defined as the randomly rewarded target) measures how strongly this overall choice strategy depends on whether the previous choice was rewarded. That is, a “win stay, lose switch” strategy would be reflected by positive weights for *T_n_*_-1_ and weights for *C_n_*_-1_ close to 0, whereas a “win switch, lose stay” strategy would be captured by negative weights for *T_n_*_-1_, and weights for *C_n_*_-1_ close to 0 (Fig. 2*c*, labels along the vertical axis). Together, *C_n_*_-1_ and *T_n_*_-1_ therefore quantify the behavioral strategies (“lose switch”, “win switch”, “lose stay”, “win stay”; Fig. 2*c*, diagonals). We examined the weights for *T_n_*_-1_ and *C_n_*_-1_ for individual sessions in the two monkeys and found that both monkeys were more likely to switch their choices, i.e., that weights for *C_n_*_-1_ were negative (Monkey 1: mean *C_n_*_-1_ = −0.371, *p* < 10^−5^, *n* = 41; Monkey 2: mean *C_n_*_-1_ = −0.159, *p* < 10^−4^, *n* = 34; average across monkeys: mean *C_n_*_-1_ = −0.275, *n* = 75; *p* < 10^−9^; Wilcoxon signed-rank tests). This tendency was slightly stronger after errors, corresponding to positive weights for *T_n_*_-1_ (mean *T_n_*_-1_ = 0.054, *p* < 0.01, *n* = 75; Monkey 1: mean *T_n_*_-1_ = 0.071, *p* = 0.017, *n* = 41; Monkey 2: mean *T_n_*_-1_ = 0.032, *p* = 0.012, *n* = 34). Intriguingly, the monkeys' highly consistent behavioral strategy to switch their choices between consecutive trials likely reflects a learned strategy to adapt to the statistics of the task. Indeed, because we presented a fixed number of stimulus presentations in a random sequence, alternating trial types were slightly more likely (∼52% rather than 50%). Note that this did not affect the 50% probability with which no-signal trials were rewarded. In Figure 3*a* probabilities for stimulus sign alternations (left) and choice alternations (middle) across all sessions are shown. For Monkey 2 the choice alternation rate (solid black line) closely matched that of the stimulus, whereas Monkey 1 overshot (dashed black line). For Monkey 2 the strategy was therefore beneficial on (weak) signal trials. Across sessions, there was no systematic relationship between the animals' choice alternation rate and that for the stimulus sign (Fig. 3*a*, right). We note that the monkeys' switching strategy contrasts with the finding in human subjects that perceptual judgments are biased toward the preceding stimulus (Fischer and Whitney, 2014). This suggests that this effect is malleable by learning depending on the task or stimulus statistics.

In control analyses using a model observer that is not influenced by trial history, we verified that our results here could not be explained by the statistics of the trial sequence (Fig. 3*b*,*c*). For such an observer the weights in Figure 2*b* would not exhibit a systematic pattern, exactly as found in our control analysis. In a subset of *n* = 50 sessions for which we had a sufficient number of segments with at least three consecutive successfully completed trials, we also explored the effect of trials going further back to trial *n*-2. We found that the additional effect was small and not significantly larger than that of choice history only including trial *n*-1 (Fig. 3*d*). We therefore restrict our analysis presented here to the effect of trial *n*-1.

### Predictive effect of spiking activity on choice

We next compared the predictive effect of choice history with that of other experimental covariates. First, we compared it to the choice-predictive effect of the spike counts of single disparity selective neurons (Nienborg and Cumming, 2006, 2007, 2009). Choice predictive effects of sensory neurons, frequently quantified as CPs (Britten et al., 1996), have been observed in a substantial number of studies, are typically modest but consistent across studies and highly statistically significant (Britten et al., 1996; Dodd et al., 2001; Uka and DeAngelis, 2004; Shiozaki et al., 2012; Nienborg and Cumming, 2006, 2014). For this comparison, we fit models incorporating different sets of parameters to all signal trials. Using the output of the models, we then predicted the monkeys' choices on no-signal trials and quantified the choice prediction performance (CPP) for the different models as the area under the ROC curve (see Materials and Methods).

Figure 2*b* compares choice prediction performance for five models. For the spike count alone, the mean choice prediction performance was 0.56 (significantly exceeding chance performance, *p* < 10^−7^, *n* = 75; Monkey 1: 0.565, *p* < 0.001, *n* = 41; Monkey 2: 0.563, *p* < 10^−4^, *n* = 34; Wilcoxon signed rank tests), very similar to the values obtained when quantifying neuron-behavior correlations as CP (Britten et al., 1996) directly from the spike count as done in previous studies (Nienborg and Cumming, 2009). This effect exceeds that of the previous target (mean CPP for *T_n_*_-1_ = 0.525, *p* < 0.001; Monkey 1: 0.519, *p* = 0.046; Monkey 2: 0.533, *p* < 0.01) but is smaller than that of the previous choice (mean CPP for *C_n_*_-1_ = 0.609, *p* < 10^−8^; Monkey 1: 0.645, *p* < 10^−5^; Monkey 2: 0.566, *p* < 10^−4^). This indicates that on no-signal trials, choice history explains a substantially larger proportion of the monkeys' choices than the single-neuron spike-count on the current trial alone. Indeed, when measuring the cumulative contribution of different covariates (the previous choice, *C_n_*_-1_, the previous target *T_n_*_-1_, whether the previous trial was rewarded, “win” *W_n_*_-1_, the spike count on the previous, *S_n_*_-1_, and current trial, *S_n_*) to choice prediction performance, choice history had the largest contribution to choice-prediction (Fig. 2*d*). For this comparison, we converted choice prediction performance into Pearson correlation coefficients and termed these “choice correlations” (Haefner et al., 2013; Pitkow et al., 2015). In this analysis, we incorporated covariates cumulatively in the model fits to account for correlations, and thus redundant information, between covariates [compare, for instance, the choice correlation of the spike count *S_n_*, when fit alone (Fig. 2*d*, red dashed line), versus the gain in choice correlation when it is included as a last covariate]. We note that since we cross-validated the model (fitting on signal trials, evaluating on no-signal trials), including more parameters does not necessarily improve performance (see Materials and Methods).

### Predictive effect of spiking history on spike counts in the current trial

**Figure 4.**
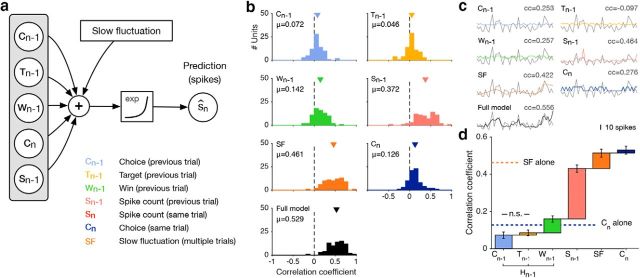
Predicting the spike count on the current trial. ***a***, Schematic showing the generalized linear model used for the analysis. ***b***, The correlation coefficients between predicted and measured spike counts on no-signal trials for models incorporating different variables across all *n* = 75 neurons. The mean values, and *p* values (Wilcoxon signed rank tests) for significant deviation from 0 were as follows: 0.072 (*p* < 10^−4^) for the choice on the previous trial (*C_n_*_-1_); 0.046 (*p* < 0.01) for the target on the previous trial (*T_n_*_-1_); 0.142 (*p* < 10^−8^) for whether the previous trial was rewarded (*W_n_*_-1_); 0.372 (*p* < 10^−10^) for the spike count on the previous trial (*S_n_*_-1_); 0.461 (*p* < 10^−10^) for Sfs across several trials; 0.126 (*p* < 10^−8^) for the choice on the current trial (*C_n_*); and 0.529 (*p* < 10^−10^) for the full model incorporating all these covariates. ***c***, Evaluating the model performance for one cell. The models were fit on signal trials and evaluated only on no-signal trials. Only no-signal trials are shown. The predicted (colored) and the measured (gray) spike counts are superimposed for each model. The performance was quantified as the Pearson's cc between the measured and predicted spike count, colors as in ***b***. ***d***, The mean correlation coefficients are plotted for models cumulatively incorporating the variables on the *x*-axis. From left to right these variables are as follows: (*C_n_*_-1_); (*C_n_*_-1_, *T_n_*_-1_); (*C_n_*_-1_, *T_n_*_-1_, *W_n_*_-1_); (*C_n_*_-1_, *T_n_*_-1_, *W_n_*_-1_, *S_n_*_-1_); (*C_n_*_-1_, *T_n_*_-1_, *W_n_*_-1_, *S_n_*_-1_, SF); (*C_n_*_-1_, *T_n_*_-1_, *W_n_*_-1_, *S_n_*_-1_, SF, *S_n_*). The height of each bar reflects the incremental improvement of the model prediction caused by the variable plotted on the *x*-axis. *P-*values (Wilcoxon signed rank tests) for the increments were *p* < 10^−4^ for *C_n_*_-1_, *p* < 10^−5^ for *W_n_*_-1_, *p* < 10^−10^ for *S_n_*_-1_, *p* < 10^−7^ for SF, and *p* < 0.01 for *C_n_*, although not significant (*p* = 0.123) for *T_n_*_-1_. Error bars are ±1 SE. Colors as in ***b***.

Spiking activity of sensory neurons is known to fluctuate on long timescales independently of the stimulus (Tomko and Crapper, 1974; Tolhurst et al., 1983; Bair et al., 2001; Ecker et al., 2014; Goris et al., 2014; Rabinowitz et al., 2015; Engel et al., 2016). Given the serial dependencies of the animals' behavior, we wanted to investigate the relationships between temporal correlations in spiking activity and behavior, as well as their impact on trial-by-trial correlations between neuronal activity and an animal's choices. We therefore first quantified the degree to which neuronal activity could be predicted by the activity on the preceding trial, to compare it to the predictive effect of choice history using GLMs (Fig. 4*a*). To additionally explore the effect of slow fluctuations (SFs) across multiple trials, we also used a Gaussian Process latent modulator (cf. Ecker et al., 2014; Rabinowitz et al., 2015) within the GLM (Fig. 4*a*). We quantified prediction performance as the correlation coefficient between the predicted and measured spike count on all no-signal trials (Fig. 4*c*; for one example unit). When fit as the only covariate, the preceding spike count had a substantial predictive effect [correlation coefficient (cc) = 0.372, *p* < 10^−10^; cc = 0.417, *p* < 10^−6^, and cc = 0.317, *p* < 10^−6^ for Monkeys 1 and 2, respectively], as expected for known non-stationarities in neuronal spiking activity. Indeed, for slow fluctuations across several trials the prediction performance improved further (SF alone: mean cc = 0.461, *p* < 10^−10^; cc = 0.477, *p* < 10^−7^ and cc = 0.442, *p* < 10^−6^ for Monkeys 1 and 2, respectively). These substantial temporal correlations of spiking activity combined with the serial dependencies of the animals' choices during this task may therefore contribute to the trial-by-trial correlations between the animal's choices and spiking activity.

### Testing for interactions between serial dependencies in choices and spiking activity

Before more directly investigating whether decision-related activity during this task results from the serial dependencies in choices and spiking activity, we examined the predictive effect of behavioral covariates on spiking activity. Specifically, we explored the effect of the animal's preceding choice (*C_n_*_-1_), the preceding target (*T_n_*_-1_), whether the preceding trial was rewarded, i.e., a win (*W_n_*_-1_) and the current choice (*C_n_*) using a GLM predicting the spike counts (*S_n_*).

Choice history had a small but significant predictive effect (the values were cc = 0.072, *p* < 10^−4^ for *C_n_*_-1_, cc = 0.046, *p* < 0.01 for *T_n_*_-1_, cc = 0.142, *p* < 10^−8^ for *W_n_*_-1_, respectively, and cc = 0.159, *p* < 10^−9^ for the covariates reflecting choice history, *C_n_*_-1,_ *T_n_*_-1,_ *W_n_*_-1_ together). When including covariates cumulatively (Fig. 4*d*) we found that only Wn-1, but not T_n-1_, yielded an improvement in prediction that was independent of *C_n_*_-1_ (note the nonsignificant change when adding *T_n_*_-1_ as a predictor, but the improvement when additionally including *W_n_*_-1_, *p* < 10^−5^). Conversely, *C_n_*_-1,_ did not improve prediction performance over *W_n_*_-1_ (*p* = 0.09, data not shown). Indeed, choice history excluding past wins (*C_n_*_-1,_ *T_n_*_-1_) was less predictive of the spike count than the choice on the current trial. Conversely, when including past wins (*C_n_*_-1,_ *T_n_*_-1,_ *W_n_*_-1_), it was more predictive of the spike count than the choice on the current trial. The predictive effect of choice history was therefore of sufficiently large magnitude to fully account for the effect of choice in principle. However, we find that a component of the predictive contribution of *C_n_* was statistically independent of choice and spiking history: even when included as the last predictor it provided a small but significant improvement in prediction performance (Fig. 4*d*; *p* < 0.01; *p* = 0.026 for Monkey 1, *p* = 0.045 for Monkey 2; Wilcoxon signed rank tests).

**Figure 5.**
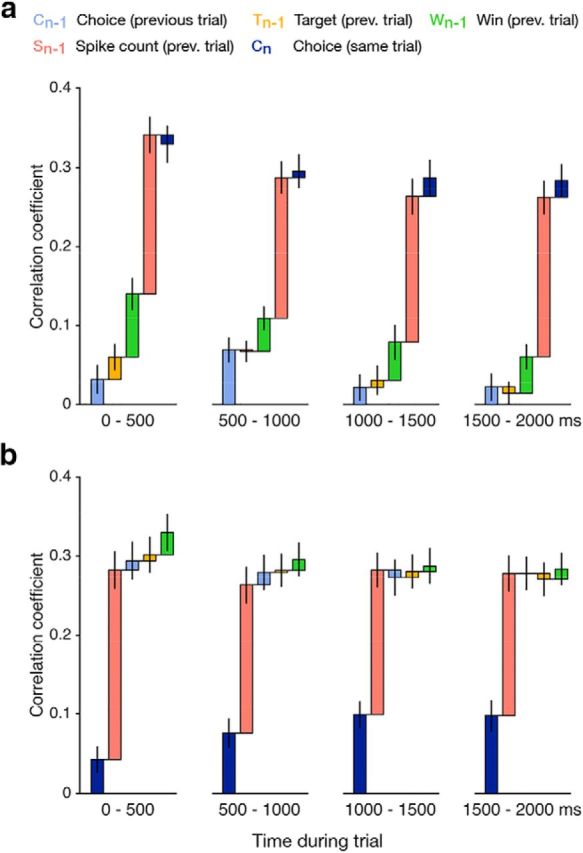
Dynamics of spike count prediction throughout the trial. For the GLM predicting time-resolved neural activity additional covariates were included: to account for the temporal structure of firing within trials, we introduced PSTH basis functions, as well as time-varying features for choice history (previous choice, previous target, previous win) and choice on current trial (see Materials and Methods). ***a***, Prediction performance across 75 units when cumulatively adding predictors for the four quartiles of the trial. The gain due to choice history decreases throughout the trial, while the gain due to choice (same trial) increases, as seen in ***b***. The grand mean prediction performance of the time-resolved model is 0.442, very similar to the prediction performance of a model that does not include additional covariates for time-resolved prediction. ***b***, Alternative order of covariates, with choice (same trial) included in the first model.

Note also the substantial component in variability that can be predicted by slow fluctuations alone (Fig. 4*d*). Interestingly, this effect is comparable to the combined predictive information in spiking-activity and behavior on the previous trial (Fig. 4*d*, compare *H_n_*_-1_, *S_n_*_-1_). This is consistent with the view that these slow fluctuations reflect meaningful signals, such as those related to choice history, as previously proposed (Rabinowitz et al., 2015). We also examined the dynamics of how these covariates predicted the neural activity during a trial. To do so, we fitted GLMs to predict the time-varying spiking activity of each neuron, and quantified the prediction performance during four nonoverlapping 500 ms wide time bins (Fig. 5). We find that prediction performance of choice history (*C_n_*_-1_, *T_n_*_-1_, *W_n_*_-1_) is most pronounced at the beginning of each trial, while the other predictors show little variation in prediction performance throughout the trial.

**Figure 6.**
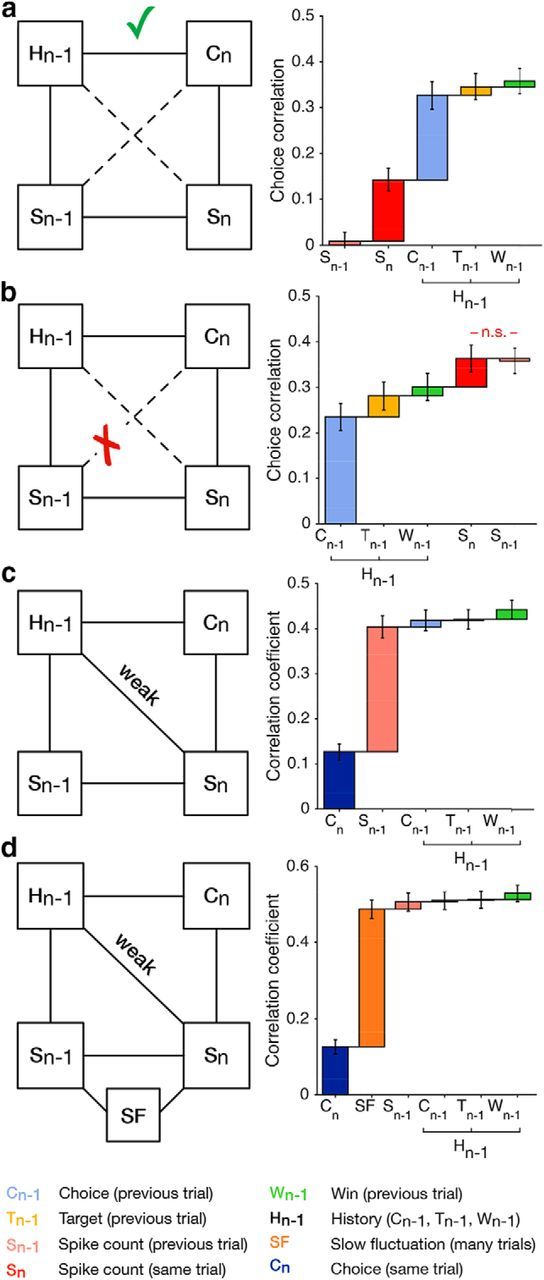
Statistical interactions between past and present choices and spike counts. Left column, Covariates are represented by the circles: spike count on the previous trial (*S_n_*_-1_), spike count on the current trial (*S_n_*), choice history (*H_n_*_-1_) and the choice on the current trial (*C_n_*). Potential (dashed lines) and statistically significant (black lines) interactions between the covariates are depicted. Right, ***a***, A model that includes choice history (*H_n_*_-1_) in addition to the previous and current spike count (*S_n_*_-1_, *S_n_*) predicts choices significantly better (*p* < 10^−9^), supporting a significant direct interaction between choice history and the current choice. ***b***, A model including S_n-1_ in addition to *C_n_*_-1_, *T_n_*_-1_, *W_n_*_-1_ and *S_n_* does not improve choice prediction performance (*p* = 0.976; Wilcoxon signed rank test). The direct interaction between *S_n_*_-1_ and *C_n_* is therefore not statistically supported. ***c***, The model incorporating choice history (*C_n_*_-1_, *T_n_*_-1,_ *W_n_*_-1_) in addition to the previous spike count (*S_n_*_-1_) and current choice (*C_n_*) predicts the current spike count weakly but significantly better (*p* < 10^−5^). ***d***, The predictive effect of choice history remains significant when including SFs (average cc = 0.506 vs 0.529; *p* < 10^−4^; Wilcoxon signed rank test).

We systematically varied the order with which we included covariates in GLMs predicting choices or spike counts (Fig. 6), to investigate which statistical interactions are necessary to explain the statistics of the data. In this visualization, potential interactions that have not been statistically evaluated are depicted by a dashed line. We then define a statistical interaction (solid connection) between a covariate and a prediction target (i.e., *C_n_* or *S_n_*) to be necessary if the predictive effect of the covariate cannot be explained by alternative covariates. For interactions that are not needed (i.e., including the covariate does not yield a significant improvement over alternative covariates) we remove the dashed connection. Note that this analysis, while related, differs from that of causal interactions in directed graphical models (see Discussion).

The substantial improvement (cc = 0.358 vs 0.143, *p* < 10^−9^) in prediction when including choice history after the preceding and current spike count (*S_n_*_-1_, *S_n_*) supports a significant statistical interaction between choice history and choice, independent of *S_n_*_-1_ and *S_n_* (Fig. 6*a*). Similarly, adding the covariate *S_n-1_* to a model that includes choice history and the current choice (*C_n_*) substantially improves the prediction of the spike count on the current trial (*S_n_*, data not shown), establishing an interaction between *S_n_*_-1_ and *S_n_*, independent of choice history and *C_n_*. Additionally, the statistically significant interaction between *S_n_* and *C_n_* (and by analogy *H_n_*_-1_ and *S_n_*_-1_, Fig. 6*a–c*) can be inferred from the results in Figures 2*d* and 4*d*: adding *S_n_* or *C_n_*, respectively, to the model that includes choice history and *S_n_*_-1_, improved the prediction of *C_n_* (Fig. 2*d*) and *S_n_* (Fig. 4*d*), respectively. (We note that for a model predicting spike counts without SF, as in Fig. 6*c*, the gain due to *C_n_* is only marginally significant: *p* = 0.036 for both animals and *p* = 0.121, *p* = 0.096 for Monkeys 1 and 2, respectively, Wilcoxon signed rank tests, and nonsignificant for paired *t* tests.)

In contrast, prediction performance is not significantly increased when including the preceding spike count (*S_n_*_-1_) after choice history and the current spike count (Fig. 6*b*). The interaction between *S_n_*_-1_ and *C_n_* independent of choice history and *S_n_* therefore lacks statistical support. Moreover, choice history provides an improvement in predicting *S_n_*, independent of *S_n_*_-1_ and *C_n_* resulting in a significant statistical interaction between choice history and *S_n_* (Fig. 6*c*). We note however that the incremental improvement in prediction is small and only owed to the predictive effect of *C_n_*_-1_ and *W_n_*_-1_. Interestingly, our time-resolved analysis reveals that this weak improvement in prediction is only present early in the trial (Fig. 5*b*). Although weak, such an effect over time is compatible with a framework of perceptual inference (Haefner et al., 2016) in which a top-down belief (or expectation) based on choice history influences the neuronal response, and is most pronounced at trial onset. Moreover, the weak increment in prediction performance resulting from *W_n_*_-1_ remains statistically significant even when incorporating the contribution of slow fluctuations, which substantially improve prediction performance (note the increased prediction performance to 0.53 of the full model in Fig. 6*d* compared with 0.44 in 6*c*). The predictive effect of *W_n_*_-1_ may therefore reflect a transient boost in arousal following a reward, leading to a modulation of the neuronal response independent of *S_n_*_-1_ and *C_n_*. Nonetheless, this effect is small, although statistically significant, the statistical interactions along the diagonal are therefore weak (Fig. 6*c*,*d*). This suggests that the serial dependencies in choices and spiking activity result from two largely separate processes. This should imply that trial-by-trial correlations between choices and neural activity are largely unaffected by these serial dependencies: CPs are a consequence of interactions on the single trial, rather than a consequence of correlations that are carried over from previous trials.

### Effect of choice and spiking history on CPs

**Figure 7.**
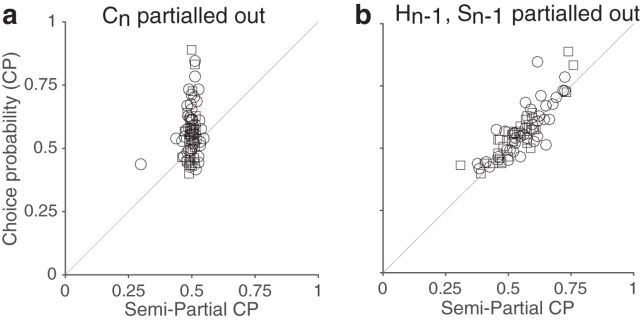
Choice probability is largely unaffected by choice history. ***a***, ***b***, We computed the residuals of the spike counts by subtracting from each measured spike count that predicted by a regression that included the current choice (*C_n_*; ***a***) or that included choice history (*H_n_*_-1_; ***b***) as covariates. In a second step, we then computed the “semipartial CPs” from these residual spike counts and the monkeys' choices on the current trial. ***a***, To verify the validity of this approach we first removed the component predicted by choice. As expected, this removes CPs deviating from 0.5. ***b***, Semipartial CPs for the residual spike counts after the contribution predicted by choice history was removed and the animals' choices, are plotted against the raw CPs (circles: Monkey 1, *n* = 41; squares: Monkey 2, *n* = 34). The values changed little and are highly correlated, supporting the view that choice probability are largely unaffected by choice history.

To test the prediction that CPs are largely independent of serial dependencies directly, we measured the residual CPs after removing the component of the spike count that could be explained by history. As choices are binary, computing and interpreting “choice residuals” by regressing out history effects is difficult; however, we circumvent these difficulties by only regressing out experimental history from spike-counts. As a result, we obtain semipartial correlation between spike counts and choice, rather than partial correlation coefficients. We found that the residual CPs were largely unchanged compared with the raw CPs (Fig. 7; *r* = 0.837, *p* < 10^−10^). Because semipartial correlations mathematically provide a lower bound to the absolute value of the partial correlations (Cohen et al., 2003), this supports the view that the contribution of choice history to CPs was at most very small. Additionally, we found that the size of the change in behavioral performance that was attributable to choice history (see Materials and Methods) was uncorrelated with choice probability (Spearman's rank *r* = 0.023, *p* = 0.848).

**Figure 8.**
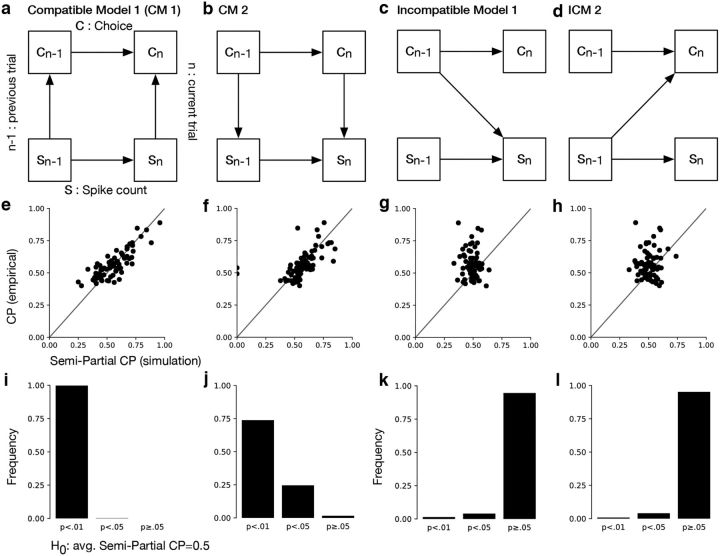
Sensitivity of semipartial correlation analysis in simulated data. ***a***–***d***, CM 1 and CM 2 are two models that are compatible with our results, whereas ICM 1 and ICM 2 are incompatible with our conclusion. We fit these models to statistics of our dataset and for each unit repeatedly simulated as many trials as no-signal trials were recorded for this unit (9522 trials across all units). ***e***–***h***, Semipartial CPs are plotted against empirical CPs but for simulated data (see Materials and Methods) for a single repetition. The data points are scattered along the identity line for the compatible models, similar to the empirical results (compare Fig. 7*b*). In contrast, semipartial CPs are reduced to ∼0.5 on average for incompatible models. ***i***–***l***, For each of 500 repetitions, we performed one-sample *t* tests with the null hypothesis (*H*_0_) that the mean semipartial CP is equal to 0.5. For nearly all of the simulations the null hypothesis is rejected for the compatible models (***i***, ***j***). As expected, we observe the opposite for incompatible models (***k***, ***l***).

Given that CPs are overall small one might wonder whether our inability to identify an appreciable contribution of serial dependencies merely results from a lack of statistical power of our analysis. To determine the sensitivity of our analysis in our dataset, we therefore repeated the semipartial correlation analysis on models that are compatible (Fig. 8*a*,*b*) or incompatible (Fig. 8*c*,*d*) with our conclusions. In the compatible models CPs reflect instantaneous, i.e., trial-by-trial, feedforward (CM 1; Fig. 8*a*) or feedback (CM 2; Fig. 8*b*) correlations between choices and spike counts. Conversely, in the incompatible models, CPs result from influences of preceding choices on current spiking activity (ICM 1; Fig. 8*c*) or of preceding spiking activity on the current choice (ICM 2; Fig. 8*c*). We fit each model to the data from each unit while matching the number of no-signal trials to those in our dataset (see Materials and Methods). Based on these model fits we then generated artificial model responses for no-signal trials and computed the semipartial CPs (regressing out choice history and spiking history). For the compatible models, the comparison of these semipartial CPs with the empirical CPs mirrors the animals' data (compare Figs. 7*b*, 8*e*,*f*). Indeed, as in our data, CPs and semipartial CPs are highly significantly correlated (average r across repetitions: *r* = 0.78 for CM 1; *r* = 0.68 for CM 2; *p* < 0.01 for all 500 repetitions for both). In contrast, for the incompatible models, choice probability is largely explained by history (Fig. 8*g*,*h*). As a consequence, CPs and semipartial CPs when removing history are not correlated (average *r* across 500 repetitions: *r* = 0.024 for ICM 1; *r* = 0.009 for ICM 2; *p* ≥0.05 in 93% and 88% of repetitions for ICM 1 and ICM 2, respectively). Moreover, semipartial CPs when removing history are 0.5 on average (Fig. 8*k*,*l*). In contrast, in compatible models we reject the null hypothesis that semipartial CPs when removing history are 0.5 in close to all cases (Fig. 8*i*,*j*). This is similar to our empirical results for which this hypothesis is rejected with *p* < 10^−6^ (*t* tests).

Together, these analyses support the conclusion that, despite the fact that serial dependencies in choices and spiking activity are both strong, they do not have a strong impact on CPs.

## Discussion

Here, we explored the effect of serial dependencies in behavior and spiking activity on decision-related activity, quantified as choice probability, of visual neurons in V2. Although the serial dependencies of both choices and spiking activity were substantial, and past choices and neural firing were correlated with both choices and spiking activity, these reflected two largely separate processes in our data: there was no statistical support for a direct interaction between the preceding spike count and the current choice, whereas the interaction between choice history and the current spike count was weak. This latter interaction resulted largely from “wins”, i.e., whether or not the preceding trial was successful and thus yielded a reward, compatible with a transient reward-dependent boost in arousal that modulated spiking activity, as well as with fluctuations in arousal that affect both firing rates and the probability of success. Importantly, when removing the effect of choice history, CPs remained essentially unchanged. The serial dependencies of choices and spiking activity therefore reflect two largely parallel processes that are correlated through instantaneous covariations between choices and spiking activity.

Our analysis based on using GLMs to determine which statistical interactions are necessary to account for the structure of the data is reminiscent of the analysis of (statistically) causal interactions in directed graphical models (Pearl, 2003). Note however that our analyses here are based on limited data such that we may not have been able to detect weak statistical interactions. In contrast, causality analyses are typically based on a “faithfulness” assumption, which states that there are no (conditional) independences other than those in the underlying graph, i.e., which interprets absence of evidence as evidence for absence. However, given limited data as in this study, such interpretation is not justified. In addition, whereas conditional dependence tests (as typically used in causality analysis) and increases in prediction performance in GLMs (as we used here) are conceptually related, they are not equivalent. In particular, our method relies on cross-validation to account for model complexity. Thus, the absence of a connection in the schematic models indicates that adding the respective covariate does not lead to a significant increase in prediction performance beyond what we can obtain by those predictors that share a direct connection.

That choice probability is independent of choice history is surprising for several reasons: first, it seems to contrast with a recent study using fMRI in humans performing a visual task that identified a signature of past choices in BOLD signals from V1 (St. John-Saaltink et al., 2016). However, apart from the different signals between studies, St John-Saltink et al. (2016) did not explore the effect of choice history independent of the current choice, which may account for this seeming discrepancy.

Second, if CPs reflect only the effect of correlated noise in the sensory representation (feedforward account), it suggests that the effect of this noise on choice is independent from trial to trial. This is interesting given the substantial slow fluctuations of the neuronal activity across trials. However, if, for example, the decision variable is computed as a difference between two pools with similar temporal correlations, then the temporal correlations could cancel when computing the decision variable, reducing or abolishing temporal correlations in the decision variable. If this is the case, it is surprising that the brain removes temporal correlations at the level of the readout of the relevant sensory information, but not temporal correlations at the decision stage.

Finally, contrasting with this pure feedforward account, CPs are increasingly thought to reflect, in part, a feedback component (Nienborg and Cumming, 2009; Wimmer et al., 2015; Haefner et al., 2016); i.e., a signal that is correlated with the animal's perceptual decision and influences the activity of the sensory neurons and thereby introduces a component of this neuron-behavior correlation. Indeed, recent work in an analogous task as the one used here supports the view that this feedback component accounts for nearly all of choice probability (Bondy and Cumming, 2018). A number of computational accounts have modeled such feedback as a signal arising from the decision variable or circuit (Wimmer et al., 2015; Haefner et al., 2016). In these models, any influence on the decision variable, such as choice history, should therefore also be fed back to sensory neurons and thus contribute to choice probability. The fact that we are unable to identify an appreciable component of choice history on choice probability therefore could imply that the feedback component to choice probability is negligible, in contrast with other findings. Alternatively, it suggests that choice history affects the decision independent of the decision-related signal that generates the feedback to the sensory neurons. Indeed, it may be that rather than relying on a single decision-variable or integrator the decision process is able to keep some influences independent. For example, the decision formation could reflect a multistage process in which a lower stage integrates the sensory evidence to form a decision variable and provide feedback to sensory neurons, and a later stage additionally incorporates the influence of choice history. Indeed, previous work identified a signature of choice history on neuronal spiking activity in the motor and frontal cortex (Pouget et al., 2011; Marcos et al., 2013), contrasting with our results for sensory cortex. It is compatible with the notion of a multistage process in which choice history affects neuronal activity only downstream of the sensory stage and of the decision circuits providing feedback to the sensory neurons. Regardless of the neuronal implementation, these results suggest that even for simple perceptual decisions models incorporating decision-related feedback require a more complex decision-formation process than current decision variables. Scrutinizing the temporal structure in neural population activity and behavior promises to provide insights into the mechanisms and computations underlying these processes.
